# *Candidozyma auris* and the Perfect Storm of Fungal Pathogenicity: Adaptation, Persistence, and Resistance

**DOI:** 10.3390/jof12040247

**Published:** 2026-03-27

**Authors:** Alessandra Vaccaro, John F. Cooper, Augusto Vazquez-Rodriguez, Hamid Badali, Ryan Kean, Gordon Ramage, Jose L. Lopez-Ribot

**Affiliations:** 1 Department of Molecular Microbiology & Immunology and South Texas Center for Emerging Infectious Diseases, The University of Texas at San Antonio, San Antonio, TX 78249, USAjesus.vazquezrodriguez@utsa.edu (A.V.-R.); hamid.badali@utsa.edu (H.B.); 2 School of Health and Life Sciences, Glasgow Caledonian University, Glasgow G4 0BA, UK; john.cooper@gcu.ac.uk (J.F.C.); ryan.kean@gcu.ac.uk (R.K.)

**Keywords:** *Candidozyma auris*, pathogenesis, antifungal drug resistance, biofilms

## Abstract

*Candidozyma auris* (formerly *Candida auris*) is an emerging multidrug-resistant pathogenic fungus with an increased ability to cause outbreaks in healthcare facilities, leading to poor patient outcomes. Since its initial discovery in 2009, *C. auris* has spread rapidly across continents and is now classified by both the Centers for Disease Control and Prevention (CDC) and the World Health Organization (WHO) as a critical-priority pathogen. This review summarizes current knowledge on the origin, taxonomy, microbiology, and virulence mechanisms of *C. auris*, emphasizing its thermotolerance, osmotolerance, and biofilm-forming capacity on biotic and abiotic surfaces, as well as aspects related to its antifungal drug resistance and management. These features, together with its genomic plasticity, contribute to persistence, transmission, and drug resistance. Emerging evidence also supports a potential link between climate change and *C. auris* evolution, highlighting environmental adaptation as a driver of pathogenicity. Combating *C. auris* will require multidisciplinary efforts to mitigate its expanding global impact.

## 1. Introduction

Humanity has been afflicted historically by the emergence and spread of infectious diseases, which remain a leading cause of death globally [1]. Among these, fungal infections (FIs) represent an increasing threat to a variety of patients. The range of FIs can vary significantly in humans, given the diverse routes of acquisition, epidemiology, tropisms, and virulence factors [2,3]. Invasive mycoses linked to these risk factors are increasingly associated with high global morbidity and mortality, with an estimated 1.7 million deaths annually [4]. Despite widespread awareness of their health implications, FIs continue to be poorly addressed. Persistent shortcomings in prevention, diagnosis, research funding, and treatment fuel the prevalence of these irrefutably important threats to our public health.

Among the causative agents of fungal infections, *Candidozyma auris* represents an increasingly significant emergent pathogen [5]. In 2019, the CDC ranked this nosocomial pathogen as an “urgent threat” due to its pervasiveness in healthcare outbreaks, its ability to form biofilms on medical equipment (e.g., catheters, hospital beds), and its multidrug resistance, which can lead to refractory treatment [3,6,7,8]. Combined, these characteristics earned *C. auris* the accolade of “superbug”. More recently, the World Health Organization (WHO) identified fungi as emergent threats to human health, with *C. auris* being classified as a high-priority organism [9]. In this narrative review, we summarize the origin, identification, biology, pathogenesis, mechanisms of antifungal resistance, and current and future directions for tackling the rapid dissemination of *C. auris*. This review reflects the expert opinions of the authors who reviewed new and relevant literature as this subject rapidly evolves.

## 2. The Emergence Hypothesis

*C. auris* was first isolated in 2009 from the external ear canal of a 70-year-old female inpatient in a Japanese hospital. As it was isolated from otic discharge, it was given the species name *auris*, derived from the Latin word for ear [3,10,11,12]. According to the CDC, *C. auris* has been detected and isolated in over sixty different countries since its first appearance, spanning all continents except Antarctica [13]. Due to its rapid spread, the CDC had stopped tracking cases in the US until its recent resurgence. It is now considered endemic in healthcare across multiple continents [14], but where did it come from?

Casadevall and colleagues hypothesized that *C. auris* did not always possess thermotolerance and halotolerance [10]. A stress-adapted *C. auris* may have risen due to natural and/or anthropogenically induced global warming, making it the first fungal pathogen to emerge due to climate change [15]. Hostile climate conditions are believed to positively select for certain stress-associated genes, and overexpression of metabolic pathways regulated by Hsp90 and the Hog1 stress-activated protein kinase (SAPK) may contribute to virulence, drug resistance, and stress resistance. These same metabolic shifts are evolutionarily conserved in *Candida albicans*. It is possible that global warming may continue to expose and spread many fungi with pathogenic potential, including *C. auris* [10].

Given its resistance to hypersaline conditions, Casadevall et al. [15] also hypothesized that *C. auris*‘s primitive environmental reservoir could have been the wetlands. These highly ionic environments exerted selective pressure, giving rise to unique adhesion factors that enhance biofilm formation in hypersaline, ionized environments, supporting the notion that this organism originates from wetlands [16]. This adaptation also promotes adherence in high salinity and lipid-rich niches, thereby facilitating persistent colonization and survival on both human skin and abiotic surfaces [17]. *C. auris* has an abrogated ability to thrive under anaerobic and highly acidic conditions [18], which infers the notion that it first existed as a plant saprophyte rather than a commensal in the mammalian gut microbiome. This may also explain why *C. auris* preferentially colonizes cooler skin sites such as the nose, groin, axilla, ear, and other skin locations with cooler temperatures rather than the bloodstream. Another interesting hypothesis as to the acquisition of virulence is the possible interspecies mating between a pathogenic *C. albicans* and a non-pathogenic *C. auris*, which may have conferred virulence to the then-saprobe via plasmid DNA transfer. This is likely given the probability that wild forms of *C. albicans* could be found in the same wetlands [10].

Selection pressure may also have led to adaptation to basal temperatures observed in mammalian and avian species, which prompted Casadevall and colleagues to conceptualize a scheme to explain *C. auris*’s migratory patterns. Briefly, after acquiring the ability to grow at temperatures between 40 and 42 °C and resistance to hypersalinity in the wake of environmental metamorphosis, adapted *C. auris* strains may have integrated into avian intermediate hosts. Migration to rural areas with human populations would quickly ensue after *C. auris*‘s use of birds as vehicular intermediaries. Moreover, the use of aviculture in rural areas may have increased fungal transmission between species, which would eventually lead to *C. auris* migration to urban areas, including hospital settings [10].

*C. auris* isolates have been exhaustively collected from various healthcare facilities across multiple continents [14], making it an exclusive human pathogen until recently. Isolation of wild *C. auris* samples from salt marshes in the Andaman Islands and from estuaries in Colombia suggested that wetland ecosystems may serve as reservoirs prior to human hosts [19,20]. Antifungal susceptibility testing found that 23 out of 24 *C. auris* samples had high MICs for fluconazole (FLU) and expected MICs for amphotericin B (AMB), while only one isolate had low MICs for both drugs. Remarkably, all isolates were obtained from salt marshes, yet 95% remained resistant. Similarly, an isolate from Colombian estuaries exhibited low MIC profiles for FLU, AMB, voriconazole, and anidulafungin [20]. In both cases, the sensitive strains were isolated from areas with sparse human activity. WGS of the Andaman samples separated the susceptible strain from the resistant strains by 77–78 SNPs, which may explain the differences in susceptibility profiles [19]. Comparative and population genomics, along with transcriptomic analyses, should be performed to identify differences in drug target genes as a means of evolution. The potential environmental reservoirs of *C. auris*, including wastewater treatment plants, plastics, and natural environments such as salt marshes, sand, seawater, estuaries, apples, and dogs, are widely discussed in the literature [21,22,23].

## 3. Microbial Characteristics

### 3.1. Phenotypic

*C. auris* colonies have a white to gray appearance on Sabouraud agar plates. Yeast cells are ovoid in shape, appearing in groups, pairs, or singles, and are typically between 2.0 and 5.0 µm in diameter [6,12]. Although budding yeast morphology predominates in *C. auris*, a study found that pseudohyphae form upon HSP90 depletion [24]. Pseudohyphal growth is characterized by a wide cell diameter compared to yeast cells, elongated cell morphology due to delayed or incomplete cell division (mother-daughter cell attachments remain), and a lack of cytoplasmic bridging and parallel divisions [25]. Growth as pseudohyphae can occur due to genotoxic stress or hypersaline conditions, and may also be triggered within the mammalian body environment [6,26,27]. *C. auris* grows well at 42 °C, and its carbon assimilation profile differs from other closely related *Candida* species, although clade- and strain-specific metabolic differences have been identified in recent years [12,28]. Moreover, *C. auris* is unable to produce chlamydospores, which are thick-walled, hyphal-specific cells produced by many fungi capable of filamentation [29,30]. When grown in fetal bovine serum, it fails to form germ tubes compared with *C. albicans*, though it retains the ability to adhere to inert surfaces [29].

### 3.2. Genotypic

*C. auris* belongs to the order Saccharomycetales within the phylum Ascomycota. Molecular analyses, including sequencing of the 26S rDNA D1/D2 domain and DNA ITS regions, place this species in the Clavispora clade of the Metschnikowiaceae family [31]. Phylogenetic relatives *Candida duobushaemulonii*, *Candida pseudohaemulonii*, and *C. haemulonii* are also part of the same family [6,32]. At least nine *Candida* spp., including *C. albicans*, *C. tropicalis*, *C. parapsilosis*, and *C. auris*, belong to the CTG clade. In this clade, CUG codes serine rather than the universal leucine, which contributes to proteomic diversity and stress adaptation [32]. This change is mediated by a mutant tRNA, Ser tRNA(CAGSer). The mutation emerged from continuous competition with its counterpart tRNA(CAGLeu) for CUG codons during the mRNA process. Eventually, this antagonism between tRNAs led to the loss of tRNA(CAGLeu) and the survival of the analogous tRNA(CAGSer), begetting the CTG clade [33]. Initial epidemiological and whole-genome sequencing (WGS) studies helped identify four karyotypically distinct *C. auris* clades grouped by geographic regions where they were initially detected: South Asian (I), East Asian (II), South African (III), and South American (IV) [34]. WGS and multi-locus sequence typing determined that the emergence of *C. auris* was simultaneous and independent across these regions. Within each clade, isolates were clonal with minimal small-nucleotide polymorphism (SNPs) diversity [6,35,36]. The reasons for the concurrent emergence of genetically distinct clades remain a mystery. The majority of infections cluster within the four main clades. Beyond these, a sample belonging to a potential fifth clade was recovered from the ear of a 14-year-old female patient in an Iranian hospital. The patient had not been exposed to other isolates from previously identified clades. WGS confirmed that the strain indeed belonged to clade V, having a marked genetic divergence of >200,000 SNPs compared to the other clades [37]. Most recently, a sixth clade (VI), also known as the Indomalayan clade, has been identified in Singapore and Bangladesh [38]. Bayesian molecular dating was used on a phylogeny of the four initial *C. auris* clades to determine the time to the most recent common ancestor (Table 1).

**Table 1:** Key characteristics and properties of the different *Candidozyma auris* geographic clades.

Clade	Primary Geographic Region of Origin	Primary Properties	Key Resistance Trends
I	South Asian	Highly invasive; often associated with large hospital outbreaks.	High resistance to Fluconazole; some Amphotericin B resistance.
II	East Asian	Often found in the ear canal (auricular); generally less invasive than other clades.	Generally more susceptible to antifungals than other clades.
III	African	Frequently associated with healthcare-associated outbreaks in South Africa.	High resistance to Fluconazole.
IV	South American	First identified in Venezuela; known for high transmissibility in ICU settings.	High resistance to Fluconazole; variable resistance to other classes.
V	Iranian	Identified more recently (2018); genetically distinct from the first four clades.	Often susceptible to most antifungals, but cases are limited.
VI	Indomalayan	The newest proposed clade (2022); identified through retrospective genomic screening.	Distinct genetic markers; research into their clinical impact is ongoing.

Retrospectively, bloodstream isolates of *C. auris*—dated back as early as 1996 in South Korea when it was misidentified by Vitek 2 YST and API 20C systems as *C. haemulonii* and *Rhodotorula glutinis*—caused the first three *C. auris*-related cases of hospital-acquired fungemia [39]. Additionally, ear isolates collected in South Korea between 2004 and 2006 that were misidentified as *C. haelumonii* were later reclassified as *C. auris* [36]. It was estimated that the current *C. auris* clades have a common ancestor dating as far back as 360 years ago, and the primary outbreak-causing clade clusters I, III, and IV appeared as early as 36 to 38 years ago [6,40]. Recently, clade V has also been associated with nosocomial outbreaks, and although clade II clusters can be sources of nosocomial spread, they are mainly associated with otomycoses [36,41]. Nonetheless, these factors all play an important role in clinical diagnostics.

### 3.3. Diagnostics and Detection

*C. auris* can often be misidentified using traditional biochemical methods. However, the use of microbiological methods such as plating clinical samples on chromogenic media (CHROMAgar *Candida*, CHROMAgar *Candida* Plus) and performing blood cultures (BioFire’s BCID 2 and the GenMark Dx ePlex BCID-FP panels) is recommended to preserve fungal cell viability and rule out other *Candida* spp. [42]. These methods are still undergoing refinement, as they may pose challenges such as cost, time-to-results, reliability, and protocol complexity, underscoring the need for affordable, rapid, and reproducible high-value diagnostic tools, especially in regions where *non-Albicans Candida* species (NCAC) are frequently reported [42,43].

Misdiagnoses are common with methods such as API 20C and VITEK2 YST ID, leading to inaccurate disease management plans [44]. Moreover, biochemical methods often require culturing yeast cells over several days, which can delay diagnosis and exacerbate disease progression [43]. Notably, the SENTRY Antifungal Surveillance Program reported no positive identification of *C. auris* between 1997 and 2016 from 135 clinical facilities in 39 countries [45]. Although *C. auris* belongs to the *Candida*/*Clavispora* clade, such as *C. haemulonii*, *C. pseudohaemulonii*, *C. albicans,* and *C. tropicalis*, it has unique characteristics that clearly distinguish it genotypically and phenotypically from the others, prompting research and development initiatives of more sophisticated diagnostic tools and optimization of currently available ones. The VITEK 2 YST ID card system is fast and dependable for the identification of yeasts and other yeast-like organisms. This is a fully automated system that employs fluorogenic methodologies and a series of carbohydrate fermentations, acid assimilations, and other biochemical reactions to determine yeast enzymatic activity. VITEK 2 can also yield results in 15 h post-inoculation using fluorescence-based methods, compared to API 20C, which requires 72 h of incubation [46,47]. VITEK 2, however, does not include methods to assess morphology, a characteristic that varies across species with and without dimorphism [46]. To avoid misidentification, further confirmation by microscopic morphological evaluation or agglutination tests may be necessary [46]. Although both systems are sufficiently reliable for identifying common pathogenic species, they are typically costly, time-consuming, and intricate, and they encounter setbacks when rare pathogens require detection [44].

Alternatively, molecular methods such as rDNA and ITS sequencing, matrix-assisted laser desorption/ionization time-of-flight (MALDI-TOF) mass spectrometry, and PCR/RT-PCR may help bridge the diagnostic gap in surveillance studies [42,43]. In fact, DNA metagenomic and metabarcoding methods based on ITS regions have been used to identify potential anthropogenic and ecological niches of *C. auris*, yielding partial matches in available metagenomic and metabarcoding databases. Some results include the possible presence of *C. auris* in Floridian peanut fields, Kuwaiti air dust, South Korean activated sludge, and even animal reservoirs such as Spanish dogs, suggesting a plethora of fungal inoculum intermediaries through which *C. auris* could have been passaged to humans [7]. The ITS regions may provide more specificity with a lower margin of error than D1/D2 domain sequencing in certain instances, particularly when misidentification is highly probable. Although D1/D2 domains are the procedural approach and can be sequenced in almost all yeasts, reference ITS region sequences are available in public databases that can be used to compare sequences between pathogenic and non-pathogenic species for more reliable results [48]. In a study where ribosomal ITS1 and ITS2 regions were thoroughly sequenced in 373 clinically important yeasts, positive agreement rates of 96.8% and 99.7% were shown, respectively [49]. Carolus et al. developed an allele-specific PCR (AS-PCR) assay targeting conserved mutations in the ITS rDNA region and a clade-specific gene cluster, enabling rapid, inexpensive, and sequencing-free identification of *Candida auris* and its major clades [50]. With recent advances in bioinformatics and automation, culture-independent RT-PCR methods such as AurisID, CanID-PCR, and FungiPlex Candida Auris have yielded promising results and shown greater advantage than conventional methods [36,51]. Moreover, promising developments have been made with Raman spectroscopy and machine-learning tools to identify *C. auris*, differentiate clades, and predict antifungal susceptibility profiles [52,53]. Together, diagnostic screening methods are critical to supporting infection prevention and control teams.

The frequency of community transmission is difficult to estimate because *C. auris* can colonize and persist at multiple body sites in immunocompetent hosts without causing visible symptoms, thereby excluding them from high-IFI risk groups [54]. As *C. auris* spreads easily in healthcare settings, the CDC recommends screening for colonization in patients with high-risk healthcare exposures [55]. It is imperative to perform comprehensive screening studies by obtaining swabs from multiple body sites in immunocompetent and immunocompromised hosts across different settings to support containment and mitigation efforts against *C. auris* [56]. It may also prove important to screen the near-patient environment where dry biofilms are known to exist [57]. Many researchers have stressed the importance of collective contributions from clinical, laboratory, public health, and government entities worldwide to reduce nosocomial spread [43]. By implementing commercial screening and surveillance methods at the local level, accurate surveying and reporting of symptomatic and asymptomatic cases can be ensured. A recent study from Baltimore detected *C. auris* in 11.81% of wastewater influent samples by qPCR and reported a weak correlation with new cases [23].

## 4. Pathogenicity

*Candida* spp. consists of over 200 species that have been taxonomically tallied for decades, with only a handful of medically important fungi [58]. The CDC reported that 95% of invasive candidiasis cases are attributable to five species [59]. *C. albicans* remains responsible for the majority of candidiasis-related IFIs, both of mucocutaneous and systemic nature, in healthcare settings, as well as the third-to-fourth most reported infection across hospitals worldwide [60]. It is closely followed by *Candida glabrata*, *C. parapsilosis*, *C. tropicalis*, and *C. kruseii* [59]. Recently, however, trends have shifted to NCAC-related infections, including those caused by the novel *C. auris*. Comparative genomics identified several genes linked to drug resistance and virulence in this emerging species compared with other *Candida* species, including expanded families of transporters and lipases, which may contribute to differences that enhance *C. auris* virulence and pathogenicity. The following subsections highlight some of the main virulence factors contributing to the pathogenesis of this emergent pathogenic yeast.

### 4.1. Morphogenetic Switching

*C. auris* differs from *C. albicans* and other dimorphic fungi in that it largely exists as a yeast. In response to atypical or unfavorable growth conditions, certain *C. auris* isolates may form pseudohyphae-like and/or even hyphae-resembling structures [36]. After passage through a mammalian host, *C. auris* exhibited a unique phenotypic switch characterized by heritable and nonheritable changes between filamentous-form, yeast cells, and filamentous-competent cells. Interestingly, some orthologs of *C. albicans* needed for attachment and filamentation, like *ALS4* and *HGC1*, were differentially expressed in the filamentous morphology of *C. auris* [27]. Notably, genomic amplification of an *ALS4* homologue (*ALS4112*) has been linked with enhanced cell–cell adherence in *C. auris* clinical isolates exhibiting aggregative phenotypes and robust biofilm formation [61]. *EFG1* and *WOR1*, which are upregulated during *C. albicans* white-to-opaque transitions appear to be downregulated in *C. auris* filamentous cells. Moreover, *C. auris* filamentous cells differentially express orthologs of *HGC1* and *ALS4*, which are involved in filamentation and adhesion, respectively, whereas factors such as *NRG1* and *CUP9* (negative regulators of filamentation) are overexpressed in *C. auris* yeast cells [27]. Genotoxic stress induced by hydroxyurea, methyl methanesulfonate, and 5-fluorocytosine can trigger pseudohyphal morphogenesis in *C. auris* Δtup1 strains, a transcription factor belonging to the family of negative hyphal growth regulators in *C. auris*. Altogether, these findings indicate that, although *C. auris* shares many evolutionarily conserved alleles with other resistant *Candida*, it has diverged in some capacity due to stress and environmental selection pressure. Genome-wide association studies are practical tools that continue to provide insight into *C. auris* copy number variants, conserved and expanded gene families and how these may impact virulence, host–pathogen interactions, and persistence [26].

*C. auris* has also been found to include strains characterized by clumping, or aggregation, and non-aggregation phenotypes that seem to have a relationship with virulence. Using a *Galleria mellonella* model, they found that the virulence of the non-aggregating phenotype of *C. auris* was similar to that of *C. albicans* and *C. tropicalis*. The larvae were inoculated with several virulent *Candida* strains, incubated at 37 °C, and dissected 18 h post-infection to reveal no *C. auris* hyphal or pseudohyphal formation compared to *C. albicans*. However, they found that non-aggregative strains of *C. auris* exhibited larval killing rates similar to those of *C. albicans* when incubated at 37 °C, whereas aggregate strains showed reduced virulence [62]. It is important to note that aggregate strains were difficult to eradicate in vitro, and regardless of larval inoculation with single yeast cells obtained from aggregates, the hemolymph-form larvae exhibited aggregation post-infection. This suggests that certain *C. auris* strains can maintain aggregation both in vivo and in vitro in physiologically unfavorable conditions. However, this does not necessarily correlate with increased virulence [62].

Despite the finding that aggregative strains are less virulent than non-aggregative strains, the former may still be associated with increased fungal burden and tissue colonization [63]. In a disseminated candidiasis mouse model comparing virulence between *C. haelumonii* and *C. auris*, immunosuppressed female BALB/c mice were challenged with both species and *C. albicans*. Results indicated that, despite prophylactic and infection-day administration of cyclophosphamide, all mice infected with *C. haelumonii* remained asymptomatic and survived for up to 12 days post-infection. On the other hand, *C. auris*-infected mice exhibited a 20% survival rate 5 days after inoculation; however, disseminated *C. auris* was not more virulent than *C. albicans* (10% survival rate by day 3 post-infection). Kidney fungal load showed a species-specific correlation between fungal burden and survival rate. *C. haelumonii* cells were not detected in culture, while *C. auris* and *C. albicans* CFUs were 5.9 × 10^4^ and 7.1 × 10^5^, respectively. Lastly, *C. auris* aggregates were ubiquitous in kidney histopathology samples, suggesting that aggregate morphology may serve as an immune evasion mechanism [64].

### 4.2. Colony Phenotypic Switching

Besides resorting to morphological switching as an immunopathogenic and virulent tactic, *C. auris* can also undergo phenotypic changes in colony morphology [6]. In *C. albicans*, the white-to-opaque switch plays distinct roles in its reproduction cycle, pathogenicity, and response to environmental stressors [65,66,67]. Like *C. albicans*, colony phenotypic switching has also been documented in *C. auris* [68]. In a study aimed at improving the classification of *C. auris* clinical isolates, *C. auris* was found to express pink, dark purple, and white colony phenotypes. Yeast cell size and shape remain invariable, and textures are smooth and glossy. In the same study, three strains across the four main *C. auris* clades were cultured in Salt Sabouraud Dulcitol-enriched broth and plated on CHROMagar *Candida* to investigate phenotypic switching and to determine inter- and intraspecies differences in this trait. Pink colonies were predominant among all clades, but clades I, II, and IV did show increased switching to dark purple colonies. Members of clades I, III, and IV are commonly associated with nosocomial outbreaks, while clade II strains are mainly associated with superficial colonization and infection. Interestingly, clade III exhibited the least amount of phenotypic switching, while clade IV had the most. When tested for switching frequency, the clade IV strain that showed the highest rate of phenotypic switching (B11245) appeared to switch preferentially between dark purple and pink colonies rather than between pink and white colonies. It is of interest that strain B11245 exhibited greater bias toward white colonies, given its increased ability to proliferate while in this phenotype. Seemingly, this predilection, which induces propagation in *C. auris* B11245, may be homologous to the increased mating affinity that opaque cells in *C. albicans* showcase upon switching [69]. On improved media such as CHROMagar *Candida* Plus, *C. auris* appears white with a surrounding blue halo. The medium also has improved sensitivity to multiple isolates, irrespective of clade or mutations, when compared against CHROMagar *Candida* [70]. This may suggest that phenotypic switching in *C. auris* is an inherited adaptive trait and could increase growth, survival, and adaptability.

Interestingly, colony phenotypic switching has been shown to correlate with ploidy plasticity in *C. auris*, particularly between pink and white colonies [71]. Cells within white colonies were smaller and had haploid genomes, whereas those in pink colonies tended to be larger in both size and genome (diploid). Transcriptomic analysis identified differentially expressed genes between diploid and haploid cells, with haploid cells exhibiting more active TCA cycle and oxidative phosphorylation than diploid cells. In addition, ploidy plasticity appears to correlate with other virulence factors such as thermotolerance, antifungal resistance, disease, and cell membrane permeability [71]. More recent studies indicate that stress factors, including temperature and available carbon sources, may drive colony phenotypic switching in *C. auris*. A recently characterized morphogenetic white-brown conversion was shown to be reversible, dependent on carbon source and temperature [68]. The study demonstrated that transitions between phenotypes correlate with shifts in genes associated with cell wall remodeling, osmotic stress tolerance, and antifungal response pathways, suggesting that switching allows the organism to better withstand environmental and host-derived pressures [68]. Overall, such factors may increase fitness and allow *C. auris* to quickly adapt to different niches, thereby increasing transmissibility.

Still, the community consensus remains that *C. auris* is haploid and that sexual reproduction via nuclear fusion and meiotic mechanisms has not been recorded. In Munoz et al.’s study, evidence supported the presence of both mating type-like (MTL) loci within all five clades in a clade-specific and homozygous manner [72]. Clades I, IV, and V are strictly MTLa, while clades II and III are MTLα. For sexual reproduction to occur, it is a requirement that strains with opposite idiomorphs come in contact. In *C. auris*, strains with differing mating loci have not been found within the same clade, but strains belonging to different clades and hence opposing mating loci have been found in different geographic locations [72]. With these findings and Casadevall’s hypothesis on interspecies horizontal gene transfer [10], sexual reproduction in *C. auris* may not be impossible. Such mechanisms would lead to increased genetic recombination and positive selection of diverse antifungal resistance genes.

### 4.3. Contextualizing the Damage Response Framework

The damage response framework, originally conceived by Casadevall and Pirofski, aimed to unify host- and microorganism-centered views of pathogenesis. It proposes that pathogenicity cannot be attributed to either the host or the microorganism alone, but rather results from interactions between them [73]. Moreover, host damage will dictate the degree of pathogenesis, and this damage could result from microbial metabolites, host responses, or both. The outcomes of these relationships are seldom simple to explain, as there is interplay between host and microbial factors that may exacerbate or attenuate benefit and/or damage for one or the other, both, or for neither. *C. auris* can be classified using this model, but because of its ability to colonize the skin irrespective of symptomology, it can be proposed that *C. auris* has the attributes of a commensal [74]. However, asymptomatic colonization by *C. auris* drives nosocomial outbreaks in hosts with weak immune systems, which can often manifest as life-threatening invasive infections. Alternatively, ear infections may occur in otherwise immunocompetent hosts, underscoring the importance of characterizing exogenous and endogenous factors that may fuel the commensal-to-pathogen transition in *C. auris*. The biology of *C. auris* can provide insight into this preferential colonization. Thermotolerance and osmotolerance are primary virulence factors that could be repurposed for adaptability on host skin. Moreover, *C. auris* can grow on biotic and abiotic surfaces, where it can persist for weeks, and has a predilection for colonizing cooler surfaces [56]. Therefore, *C. auris* could be a chronic colonizer of human skin, where it may micro-diversify and adapt to already present ecological consortia, establish mutualistic or commensal polymicrobial interactions with other fungi or bacteria on the skin, and trigger mild to modest immune responses that allow it to remain in homeostasis, much like *Malassezia* spp. The skin is also a source of lipids, lipid-producing enzymes, and carbohydrates that can ease the onerous task of nutrient scavenging in unfavorable environments [75]. It may be possible that in a comfortable niche constantly exposed to environmental factors such as the skin, *C. auris* could undergo transcriptional and epigenetic changes that enable optimal adherence, metabolic shifts, and survival. Fungi also grow well at varying pH levels, and given that the skin’s pH is generally acidic, *C. auris* may adapt and grow well under these conditions while other possible *C. auris* predators may be unwelcome [76].

Beyond the skin barrier, *C. auris* can invade and become systemically important [77]. In an in vivo murine model of disseminated candidiasis, *C. auris* was reported to be less virulent than *C. albicans*. After challenging immunocompetent C57BL/6J mice with the same colony-forming unit (CFUs) loads intravenously for both *C. albicans* and *C. auris*, survival rates were lower for *C. albicans* infection compared to *C. auris*. Likewise, they demonstrated decreased fungal burden in the kidneys of *C. auris*-infected mice after 7 days compared to organs infected with *C. albicans*. Importantly, results correlated with better immunomodulation and antifungal defense against *C. auris* in immunocompetent hosts. This was demonstrated by *C. auris*’s ability to induce a more potent immune response upon stimulation with patient-derived peripheral blood mononuclear cells (PBMCs). After 24 h, PBMC exposure to *C. auris* resulted in broader transcriptional responses than those observed with *C. albicans*, with several unique *C. auris* differentially expressed genes correlating with enriched host responses, emphasizing cytokine production and certain interferon responses [78]. Furthermore, myeloperoxidase production in immunocompetent mice following *C. auris* and *C. albicans* infection was markedly robust, contradicting prior findings of impaired human neutrophil recruitment and neutrophil extracellular trap formation against *C. auris* relative to *C. albicans* [79]. Despite these observations, *C. auris* is more likely environmentally adapted and should be considered an accidental pathogen. This is why it can persist, colonize the skin, and survive effectively within the healthcare environment.

### 4.4. Skin Colonization and Persistence

The persistence of *C. auris* on patients’ skin poses a major concern to all healthcare professionals. Unlike other *Candida* species that primarily inhabit mucosal membranes, *C. auris* shows a pronounced adhesion to and a predominant tendency for skin colonization [7,36]. Its prolific capacity for skin colonization and persistence accelerates its dissemination across the healthcare setting, as skin cells are constantly shed from infected patients, creating reservoirs for further transmission [76,80].

**Figure 1:**
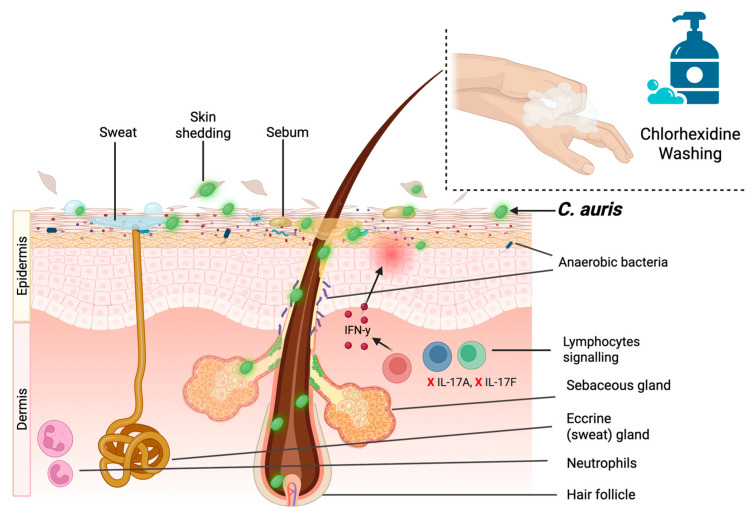
Host and cutaneous environmental factors promoting *Candidozyma auris* colonization, persistence, and dissemination from the human skin. The epidermal microenvironment provides conditions favorable to *C. auris*, including moisture from sweat and lipid-rich sebum, which may support metabolic flexibility and utilization of alternative carbon sources. Continuous desquamation of corneocytes contributes to the mechanical dissemination of *C. auris* onto surrounding environmental surfaces. Disruption of the resident cutaneous microbiota, including through chlorhexidine washing, may reduce microbial competition and promote *C. auris* persistence. *C. auris* can localize to protected niches such as hair follicles and associated sebaceous structures, potentially limiting exposure to topical antiseptics. Within the dermal/epidermal interface, interactions with immune cells are depicted, including neutrophils and lymphocytes. Evidence suggests altered neutrophil engagement and the induction of interferon-γ (IFN-γ)-associated responses, which may contribute to local inflammation and compromise epithelial barrier integrity. Reduced or dysregulated IL-17A and IL-17F signaling is illustrated, consistent with impaired antifungal mucocutaneous defense pathways. Together, these factors create a permissive cutaneous niche that supports *C. auris* colonization, persistence, and transmission. Created in https://BioRender.com.

*C. auris* has been found to enrich populations of certain resident skin cells like type 3 innate lymphoid cells, γδ cells, and IL17A, IL17F Th17 and Tc17 cells upon skin colonization. In fact, it preferentially lodges within deep skin tissue sections, where glands and hair follicles are found (Figure 1). This may be another adaptive mechanism for immune evasion, as host immune effector cells cannot readily reach those areas, concomitant with nutrient scavenging [81]. Colonized patients often exhibit no symptoms and can remain positive for long periods, with one surveillance study finding the median time for patients to become serially negative from an initial positive screen was 8.6 months (IQR 5.7–10.8 months) [80,82]. Further research found that *C. auris* remained within the follicles and sebaceous glands of murine skin months after skin surface screening was negative [83]. This explains how *C. auris* can proliferate on patient skin despite frequent chlorhexidine gluconate (CHG) washing, as the compound lacks permeation into the follicles and glands where the pathogen resides [80,84]. Recent studies, however, have shown that augmenting CHG with isopropyl alcohol or certain essential oils greatly improved decolonization in vitro and ex vivo [85,86].

Several factors give *C. auris* an advantage over other *Candida* species when colonizing the skin. The adaptation of several species-specific adhesion factors may play a prominent role in biofilm formation, skin colonization, and virulence. The *C. auris*-specific Surface Colonization Factor (*SCF1*) differs from the majority of fungal adhesins by utilizing cation-substrate interactions that enhance long-term skin adherence and biofilm formation [16]. The identification of a *C. auris*-specific *IFF* homolog (*IFF4109*), which is involved in skin surface adhesion and virulence, was also an important discovery [16]. In addition, the *ALS* homolog (*ALS4112*) has been shown to convey robust adherence to host ECM proteins and keratinocytes in vivo [87]. Further investigation using an in vivo murine model found that deletions of the adhesin genes *SCF1* and *ALS4112* significantly affected cell–cell adherence and biofilm formation depending on the aggregative growth phenotype [88]. This functional diversity of cell wall adhesins provides insight into how *C. auris* strains have adapted to effectively colonize and persist on the skin surface. A recent study found that Hog1 mitogen-activated protein kinase is essential for regulating cell wall organization and the expression of cell adhesins, including *IFF4109*, *ALS5,* and a multitude of other uncharacterized adhesins involved in biofilm formation and skin adherence in vitro and ex vivo [89]. The nature of *C. auris*-host interactions is complex and understudied, with a multitude of skin-specific adhesion factors yet to be characterized [88,89]. Furthermore, *C. auris* has specific immune-evading properties and interactions with commensal skin microbiota that remain poorly understood [74]. Low neutrophil recruitment and a lack of IL-17A and IL-17F signaling by innate and adaptive lymphocytes have been associated with long-term *C. auris* skin colonization and persistence in immunocompromised individuals [79,83]. There is also evidence supporting a link between skin microbiota dysbiosis and *C. auris* colonization and persistence, with microbiome dysbiosis a common factor among *C. auris*-positive patients [90]. Microbiome dysbiosis is inevitable in patients receiving frequent CHG washes, possibly exacerbating *C. auris* persistence in immunocompromised patients [83]. Herein lies the value of in vivo and ex vivo studies, which help elucidate the mechanisms underlying skin persistence and inform the formulation of long-term decolonization strategies.

Current data highlight the widespread use of CHG at 2–4% in wipes and baths as a primary measure against *C. auris* colonization [83,85,91]. However, there is concern about its limited permeation into the skin, which correlates with lower reductions in fungal load and long-term persistence [86]. Using an ex vivo porcine model, it was shown that the efficacy of CHG/isopropanol is enhanced when combined with natural antiseptics, such as tea tree and lemongrass oils, for decolonization [86]. Follow-up studies are necessary to assess the efficacy of improved CHG formulations in humans. Other natural antiseptics, such as manuka honey, eucalyptus oil, and thymol, have also shown promise in reducing *C. auris* fungal burden, while synthetic compounds containing povidone-iodine, octenidine, and chlorine are most effective in vitro [92,93]. Additionally, research into liposomal technology to improve the delivery of novel and repurposed antifungals may lead to a breakthrough in effective *C. auris* decolonization and treatment [94,95]. Recent work has described a carbonic sensing pathway (CSP) that contributes to amphotericin B resistance, but, through detailed integrated omics approaches, has shown that the carbonic anhydrase Nce103 is an important regulator. It is hypothesized that bacterial urease activity, which releases CO_2_, improves *C. auris* fitness on the skin [96]. This knowledge may support opportunities for therapeutic exploitation. Despite these experimental breakthroughs, there is currently no approved strategy for decolonization of infected patients, meaning that without active screening programs, colonized individuals may inadvertently spread across sensitive hospital areas. To address this problem, understanding of the factors regulating *C. auris* skin colonization and persistence in vivo is vital for developing effective decolonization strategies for infected patients.

### 4.5. Biofilm Lifestyle: Impact on Pathogenesis and Clinical Repercussions

Observations from ex vivo studies and animal models have demonstrated that *C. auris* can form biofilms in synthetic sweat media and persist in nutrient-poor, high salinity skin conditions [17,97]. Ongoing research suggests that *C. auris* employs alternative carbon metabolism, enabling it to use sweat and sebum components as nutrient sources [28,75,98]. High-throughput profiling identified unique genes within the *C. auris* clades, enabling robust growth on alternative carbon sources and on dipeptides as a nitrogen source [28]. These *C. auris*-specific metabolic genes may explain its robust growth and persistence observed in the axilla and groin of patients [82]. It was also demonstrated that the presence of artificial sweat and sebum enhanced *C. auris* biofilm formation on porcine skin [17]. The findings showed that the fungal burden of *C. auris* was 10 times greater than that of *C. albicans*, indicating that *C. auris* can thrive as a biofilm in high-sweat body sites, which correlates with clinical data [17,76,82].

**Figure 2:**
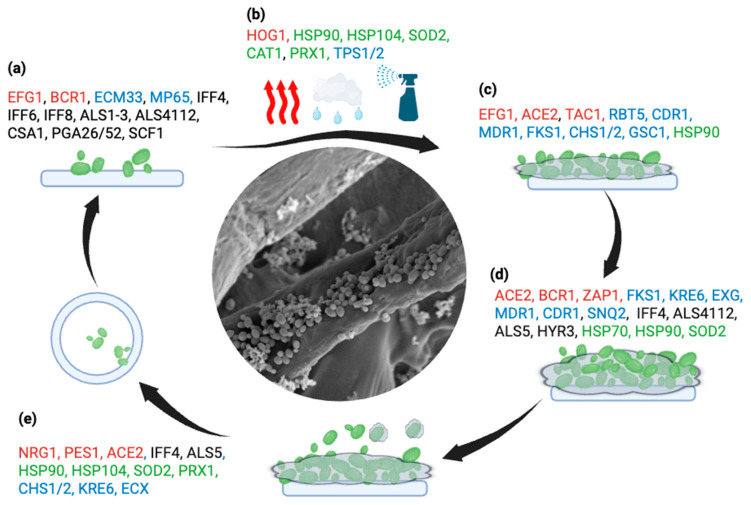
Schematic representation of phase-enriched transcriptional programs underlying sequential stages of *Candidozyma auris* surface colonization and biofilm development. Schematic representation of key transcriptional patterns identified during each stage of *Candidozyma auris* surface colonization and biofilm development. Transcriptional Regulators/Signaling (Red), Adhesion Factors (Black), ECM/Cell Wall/Matrix Regulators (Blue), Stress Adaptation Factors (Green). During initial attachment (**a**), adhesins including IFF4/6/8, ALS1/4, CSA1, PGA26/52, and SCF1 facilitate surface adherence under the regulation of EFG1 and BCR1, with cell wall organization supported by ECM33 and MP65. The response to drastic changes in heat (red arrows), osmotic pressure (rain cloud), and exposure to disinfectants/oxidizing agents (spray bottle) is depicted in the stress adaptation phase (**b**). HOG1-mediated signaling and induction of stress response genes (HSP90, HSP104, SOD2, CAT1, PRX1) alongside metabolic regulators (TPS1/2) promote environmental tolerance. Persistence and early biofilm formation (**c**) involve regulatory activity of EFG1, ACE2, and TAC1, alongside cell wall and efflux-associated genes (CDR1, MDR1, FKS1, CHS1/2, GSC1) and sustained HSP90 expression. During biofilm maturation (**d**), ACE2, ZAP1, and BCR1 coordinate matrix development and intercellular adhesion, with contributions from FKS1, KRE6, EXG, CDR1, SNQ2, and RBT5. In dispersal (**e**), regulatory shifts involving NRG1, PES1, and ACE2 accompany expression of adhesins (IFF4, ALS5), cell wall remodeling (CHS1/2, KRE6, EXG), and stress tolerance genes, priming released cells for survival and reattachment. Created in https://BioRender.com.

*C. auris* is much like other *Candida* spp. by virtue of its ability to persist as a biofilm, much like other medically relevant fungal species. Biofilms are characterized by a dense consortia of adherent sessile cells that can form tightly woven, heterogeneous communities of multi-morphic cells embedded in an extracellular matrix (ECM). This enables them to adhere to biotic and abiotic surfaces [60,99]. As such, these highly organized microniches further complicate systemic, dermal, and mucosal infections. In yeasts such as *C. albicans*, biofilm formation is a highly regulated process that proceeds through four major steps: adherence, proliferation, maturation, and dispersa [100]. Biofilm biogenesis relies on nutrient availability, sensory mechanisms, positive and negative transcriptional regulation, and divergent gene expression profiles that can induce metabolic and adaptive changes optimal for nutrient acquisition and survival [99]. Figure 2 illustrates the key phases of biofilm development and the accompanying transcriptional changes.

Although *C. auris* biofilms are substantially weaker than those of *C. albicans*, they have a persistent ability to adhere to both skin and abiotic surfaces [101]. Phenotypically, *C. auris* biofilms differ in that they do not contain true hyphae, but rather clumpy aggregates or single yeast cells with occasional pseudohyphae [6,29,30,36,102]. Biofilm spatial architecture largely depends on whether the strain is aggregated or non-aggregated. Vila et al. showcased fluorescent confocal images of *C. auris* isolates 0382 and 0387 belonging to the CDC AR-Bank. Isolate 0382 is classified as a high-biofilm former as opposed to isolate 0387, with the former exhibiting a dense and homogenous multilayer, while the latter formed a dispersed biofilm with scattered cell aggregates. Both presentations were synonymous with aggregate and non-aggregate *C. auris* phenotypes, respectively [103]. Interestingly, both biofilm subtypes are associated with increased secretion of exopolymeric materials into the biofilm matrix, a finding not supported by studies on a silicone elastomer [29]. Vila et al. also developed an oral infection mouse model in which *C. auris* did not colonize the tongue dorsum of infected mice, whereas *C. albicans* formed robust biofilms, with hyphae penetrating and damaging epithelial tissue [103]. A further study of histatin 5, a cationic peptide found in saliva, has demonstrated potent inhibitory activity even against FLU-resistant *C. auris* isolates [104]. Given that mice cannot produce this peptide, it can be surmised that other factors prevented colonization.

Transcriptional differences exist between *C. albicans* and *C. auris* biofilms, which define their predilections for colonizing tissue in vivo and ex vivo and attaching to certain surfaces. Such heterogeneity may influence mechanisms of drug tolerance and sensitivity. Several virulence genes that encode glycophosphatidylinositol (GPI)-linked cell wall adhesins, integrins, and agglutinin-like sequences become upregulated during biofilm formation and maturation in *C. auris*. Interestingly, seven of those upregulated GPI-anchored proteins (*IFF4*, *PLB3*, *PGA52*, *PGA26*, *CSA1*, *HYR3*, *PLB3*, and *PGA7*) are evolutionarily conserved between *C. auris* and its phylogenetic relatives *C. haelumonii*, *C. pseudohaelumonii*, and *C. duobushaemuloni* [32,36,72]. Transcriptomic analysis of in vitro grown *C. auris* biofilms revealed the presence of ALS1 and ALS5 adhesins, but not ALS3, a key adherence factor in *C. albicans* involved in host cell endocytosis [105]. Other putative virulence factors like lytic enzymes, which are critical for morphogenesis, adherence, and invasion, such as hydrolases, hemolysin, secreted aspartic proteinases (SAPs), and phospholipases have also been documented in *C. auris* [27,30,32,72]. Different phenotypic switches induced by temperature in *C. auris* are also correlated with changes in SAP activity [102]. Together, these data indicate that the biofilm lifestyle is critical to *C. auris*, with significant implications for its clinical management due to the tolerant phenotype associated with biofilms.

## 5. Antifungal Drug Resistance

*C. auris* has been called a “superbug” due to its synchronous geographical appearance, territorial distribution, tendency to cause hospital outbreaks, and quick multi-drug resistance evolution. All of which reinforces the global threat it represents. Its pan-resistance can be traced back to its phylogenetic relationships with other highly resistant NCAC species. Population genomics analyses found that four strains belonging to Clade I exhibited resistance to all antifungal classes used to treat systemic infections, underscoring the need to devise strategies to mitigate resistant phenotypes [40].

Antifungal resistance in pathogenic fungi is a complex, multifactorial phenomenon that cannot be attributed to a single specific factor. Intrinsic resistance, stemming from an absence of drug pressure, is exemplified by the fact that 90% of global *C. auris* isolates exhibit intrinsic resistance to FLU [36]. This innate resistance commonly reflects specific physiological traits, including decreased drug binding due to target alteration, upregulation of efflux pumps, or altered drug permeability rates [106]. *C. auris* tolerance and resistance mechanisms to the three main antifungal classes are discussed and summarized (Table 2):

### 5.1. Azoles

*C. auris* exhibits an intrinsic resistance to triazoles, likely due to its phylogenetic relationship to other intrinsically resistant species coupled with extensive azole use in fields and clinics [7,36]. Azoles target lanosterol 14-α-demethylase, an important enzyme in the ergosterol synthesis pathway encoded by *ERG11*. Mechanisms of triazole desensitization include overexpression of the target enzyme, mutations in *ERG11* that may alter azole affinity for the target, and increased efflux pump activity [6,106]. FLU seems to be the main target for resistance and tolerance, given that it is the most frequently administered and readily available antifungal in an array of conditions. Prophylactic and empiric administration may lead to drug-induced selection pressure, which can open the door to hypermutation, loss of heterozygosity, and increased mutation rates that confer resistance or tolerance (e.g., point mutations, amino acid substitutions, insertions, etc.) [106,108]. For instance, *Candida* spp. have been found to possess hotspot mutations in the *ERG11* alleles, including amino acid substitutions at Y132F, K143R, and F444L, all of which confer azole resistance. Because the *C. auris* genome has been sequenced, screening studies have found the same variants along *ERG11* [7,106,107,112]. A study by Healey et al. found a two-fold azole MIC increase upon insertion of Y132F or K143R substitutions in a *Saccharomyces cerevisiae* recombinant strain compared to a strain only expressing the wild-type *C. auris ERG11* gene, suggesting decreased azole sensitivity in the presence of these allelic expressions [113].

Using a specialized Cas9-ribonucleoprotein transformation system, it was found that deleting the *CDR1* homolog in *C. auris*, which encodes an efflux pump, increased azole susceptibility in an azole-resistant strain by 64–128-fold [108]. More recently, it was shown that mutations in *TAC1B*, which encodes a zinc cluster transcription factor, conferred increased resistance to azoles by upregulating *CDR1* and *MDR1* [109]. Multiple mechanisms of azole resistance related to physiological changes may also be present in *C. auris*, much like in other *Candida* spp. For instance, Hsp90 expresses the Heat Shock Protein 90 (HSP90), a chaperone protein that aids in stabilization of proteins during heat- and drug-induced stress, protein folding, and even heat-dependent morphogenetic changes in pathogenic *C. albicans* strains. In *C. auris*, blockage of Hsp90 using a doxycycline-repressible promoter resulted in abrogated azole tolerance, but strains possessing *ERG11* mutations and a Hsp90 depletion remained unaffected. Depletion of the former seems to be responsible for filamentation in *C. auris* as well. The same group found that FLU resistance could also be mediated via *CDR1* overexpression, independent of Hsp90 [24].

### 5.2. Polyenes

Resistance to polyenes, such as Amphotericin B (AMB) and nystatin, is rare across fungal species because of their fungicidal properties. Polyenes target the main component of the fungal cell membrane: ergosterol. The latter is not a translation byproduct; therefore, it is not considered a protein target [110]. Polyenes will form a complex with ergosterol in the cell membrane, leading to the formation of porous channels that cause ion leakage, destabilize the concentration gradient, and ultimately cell lysis [106,114]. If resistance does occur, it is often due to alterations in ergosterol biosynthesis in response to cell membrane stress that may lead to either upregulation of *ERG* genes or loss-of-function mutations that reduce membrane ergosterol levels. The exact mechanisms of resistance and tolerance to AMB in *C. auris* remain elusive, with some studies suggesting genetic expansion or intrinsic resistance, while others suggest metabolically transient pathways or involvement of the transcriptome. Worryingly, AMB resistance may also arise during treatment [7,106,112]. For instance, several studies have reported high AMB MICs across a range of *C. auris* isolates from India, Russia, the USA, and Colombia. The consensus is that *C. auris* AMB resistance ranges from 0 to 30%; however, conservative studies suggest an overestimation, and a figure of 12% is more accurate [115,116]. Since *C. albicans* notably exhibits amino acid substitutions in *ERG2*, *ERG3*, *ERG5*, *ERG6,* and *ERG13* that can alter sterol composition in response to polyenes, it is hypothesized that *C. auris* may employ a similar mechanism, but further studies are required [112,113]. Interestingly, different genome-wide studies found that *C. auris* intrinsically overexpresses ABC- and MFS-type multidrug transporter gene orthologs, which encode efflux pumps that contribute to virulence and resistance in *C. albicans* [107]. To further complicate matters, definitive clinical breakpoints have not been established by the Clinical Laboratory Standards Institute (CLSI) or the European Committee on Antimicrobial Susceptibility Testing (EUCAST) due to the large genomic variability and, consequently, susceptibility profiles among clades (Table 2). Although facsimile patterns were observed for FLU, no definitive parameters could be established for other antifungal classes [36,110].

### 5.3. Echinocandins

The mechanism of action (MOA) of echinocandins is focused on the uncompetitive inhibition of β-1,3-glucan synthase, the major enzyme involved in the synthesis of β-glucan, a major structural component of the fungal cell wall [117]. Glucan, along with chitin, intertwines with other major cell wall components, such as galactomannans and mannoproteins, conferring rigidity and protection to the cell wall while aiding in the preservation of cell membrane integrity [118]. Echinocandin resistance in *C. auris* is observed at lower rates than those of azoles and polyenes, but there have been increasing reports of patients becoming refractory to echinocandin therapy during treatment [107]. According to the CDC, less than 5% of *C. auris* USA isolates are echinocandin-resistant. Although rare, it is not uncommon to expect resistance rates to continue to rise as *C. auris* infections are reported worldwide, especially in regions where individuals may have been previously treated with echinocandins [107,110]. In *Candida* spp., molecular methods have helped identify hotspots in the *FKS1* and *FKS2* genes, termed HS1 and HS2, both of which encode the subunits of β-1,3-glucan synthase. In the case of *C. auris*, only *FKS1* amino acid substitutions have been identified at S639F/Y/P positions, which also align with HS1 in *C. albicans FKS1* [7,36,106]. S639F conferred echinocandin pan-resistance to 4 isolates in a study where 106 *C. auris* isolates were sequenced and tested for antifungal susceptibility [119]. Several other mutations in the *FKS1* gene have been identified more recently, associated with increased resistance to echinocandins [120]. These mutations can now be searched using a new benchmarking tool containing 100 characterized WGS strains [121].

Very few *C. auris* strains are intrinsically resistant, making echinocandins first-line treatments in the event of a *C. auris* infection [6,36]. However, therapeutic failure is likely if echinocandin-resistant or pan-resistant *C. auris* isolates are encountered, much like in cases where *C. albicans* was exposed to echinocandins for prolonged periods during esophagitis [110]. In addition, we note that tolerance and heteroresistance to echinocandins in *C. auris* have recently been described, although their clinical significance remains to be determined due to the limited data correlating them with treatment failures [111]. Rising broad-spectrum resistance, combined with fungal pathogens that harbor intrinsic echinocandin resistance (e.g., C. *neoformans*), urges the need to expand the current antifungal armamentarium [122].

## 6. Future Therapeutic Directions

The first step in combating antifungal resistance and tolerance is to use methods to screen for and identify resistant strains and their associated determinants. Surveillance and epidemiological studies have been essential for characterizing microorganisms that are unresponsive to currently available therapies, and organizations such as CLSI and EUCAST have established standardized protocols for in vitro susceptibility testing, which are available directly from the CDC and EUCAST [123,124]. Methods include broth microdilution, disk diffusion, strip-based Etest, and azole agar screening. While these reference methods are considered the gold standard in medical mycology for establishing clinical cut-offs (Table 3), many breakpoints in understudied species are tentative or nonexistent. These parameters need to be evaluated across all clinical isolates being tested, as species-specific variability can pose reproducibility and sensitivity issues [6,36,44,110]. Additionally, breakpoints have been established for only a handful of commonly occurring dimorphic fungal pathogens (e.g., CLSI M27-A2 and M38 for yeasts and filamentous fungi) using only the main antifungal agents. Because of protocol variation, EUCAST and CLSI results are difficult to compare, and certain unculturable species cannot be tested [110]. Moreover, these standardized methods are limited to planktonic cell susceptibility, and many pathogenic fungi form biofilms [125]. More recently, novel automated methods such as the MALDI Biotyper antibiotic susceptibility test rapid assay (MBT ASTRA), based on MALDI-TOF mass spectrometry, and isothermal microcalorimetry (IMC) show promise as future susceptibility testing methods [126,127]. Nevertheless, MALDI-TOF MS requires highly skilled personnel for operation, and equipment costs may be too demanding for smaller facilities [43,44]. IMC may be a cost-effective alternative, as it has shown reliability when tested on biofilm and planktonic conditions in *C. auris* and compared against other available metabolic signature testing methods, such as VITEK2 [127]. Limitations still linger; hence, further validation studies are required to determine the extent of reliance on use in surveillance studies.

For *C. auris*, official clinical susceptibility cut-offs have not been established, but the CDC suggested tentative breakpoints based on common pathogenic *Candida* spp. and expert opinion for all major antifungals in drug-resistant strains [36]. The MIC breakpoints are summarized in Table 3. Given that >90% of *C. auris* strains are intrinsically resistant to FLU, which has the same MOA as other triazoles, MICs have not been established for second-generation triazoles, as susceptibility profiles vary across isolates [128].

Genes encoding fungal drug targets and mutations conferring resistance can be identified using molecular methods such as sequencing, comparative genomics, RNAseq, and RT-PCR. Although some of these techniques can be used in laboratory and clinical settings with minimal training due to their improvements in specificity, sensitivity, and ease of use, DNA sequencing remains the preferred method for detecting resistance, which limits its clinical use given the expense of sequencing machinery [43,110]. Given that resistance and tolerance may have a polygenic and pleiotropic nature coupled with functional redundancy, developing more targeted methods together with the evolution of bioinformatics, techniques like spatial transcriptomics, in silico studies, nanopore sequencing and single cell RNAseq may be used to obtain a thorough outlook of drug-target interactions, tolerance and resistance pathways in the real space continuum, which could aid researchers in the drug discovery process.

High-throughput screening (HTS) of chemical libraries is an attractive method for identifying compounds with antifungal activity and can be performed using in vivo and in vitro models coupled with robotics and software [130]. For instance, protocols have been developed for HTS against *Candida* biofilms using 96-well and 384-well microtiter plates [131,132]. The protocols are simple, reproducible, cost-effective, rapid, and easy to implement across small and large laboratory settings. They are coupled with an XTT 2,3-Bis-(2-Methoxy-4-Nitro-5-Sulfophenyl)-2H-Tetrazolium-5-Carboxanilide reduction color-imetric assay, whereby water-soluble XTT is converted by actively respiring cells into a water-soluble, orange-colored formazan product [131,132,133]. Results can be read spectrophotometrically using a microtiter plate reader, whereby metabolically active sessile cell activity has a linear relationship to cell density and, subsequently, signals are detected from absorbance readings. HTS has also been used to identify compounds with repositioning potential. These compounds will generally have a high safety profile because they are already FDA-approved and have well-characterized mechanisms of action and ADMET properties. This makes drug repurposing an attractive solution to high attrition rates and time-consuming research and development facing the pharmaceutical industry [134].

Alternative strategies, such as screening the non-exhaustive natural repertoire of bioactive compounds, including phenolic compounds, and even synthesizing silver nanoparticles with natural antioxidant and antivirulence properties, have shown great promise and continue to be adopted globally in the war against multidrug resistance [8,106]. Combination therapy approaches have also proven successful, with classic antifungals administered in tandem to achieve synergistic and/or additive effects. For example, the monoterpene carvacrol (CAR) was combined with AMB, FLU, nystatin (NYS), and caspofungin (CAS) in an in vitro screen of *C. auris* virulence factors using buccal epithelial cells. They found that CAR exhibited anti-adherence and anti-proteinase activities against *C. auris* at subclinical concentrations, without inducing pseudohyphal morphogenesis or penetration into epithelial cells. Furthermore, CAR-AMB and CAR-NYS combinations resulted in synergy across 28% of the tested strains, of which 12% and 28% of the 25 total strains were resistant to both antifungals, respectively. Additivity was also observed in 52% and 24% of strains in the CAR-FLU and CAR-CAS combinations, among which 48% were FLU-resistant, and none were CAS-resistant [135].

Prospective directions in antifungal therapy may include fungal vaccines and immunotherapy, which require an in-depth understanding of innate and adaptive responses in fungal immunity and careful safety considerations to avoid excessive reactogenicity and inflammatory dysregulation in immunocompromised hosts, sometimes referred to as cytokine storm [110]. Although no fungal vaccines have been licensed to date, several attempts have been made, with some, such as the *C. albicans*-targeted NDV-3, advancing to phase II clinical trials [136].

## 7. Conclusions and Outlook

*C. auris* has rapidly established itself as one of the most formidable fungal pathogens of the 21st century. Its remarkable adaptability, thermotolerance, osmotolerance, capacity to persist on biotic and abiotic surfaces, and transmissibility have fueled its global emergence as a multidrug-resistant healthcare-associated threat. The convergence of virulence factors, antifungal resistance, and unique ecological versatility underscores the urgent need for continued surveillance, improved diagnostic tools, and coordinated infection control efforts. Understanding *C. auris* within the broader context of fungal pathogenesis highlights the intricate balance between microbial adaptation and host defense that determines disease outcomes.

Despite major advances in our understanding of *C. auris* biology and resistance mechanisms, significant knowledge gaps remain regarding its environmental reservoirs, transmission dynamics, and host immune interactions. The emergence of pan-resistant strains and the limited antifungal pipeline demand innovative approaches that combine drug repurposing, novel antifungal development, and immunotherapeutic strategies. Integrating high-throughput screening technologies with genomic and transcriptomic analyses will be instrumental in identifying new therapeutic targets and resistance determinants.

Ultimately, the story of *C. auris* serves as both a scientific and public health warning. Its rise illustrates how environmental change and global interconnectedness can converge to drive the emergence of new pathogens. Moving forward, a multidisciplinary approach that unites microbiology, immunology, ecology, and clinical medicine will be essential to mitigate the ongoing threat posed by *C. auris* and to strengthen preparedness against future fungal epidemics.

## Figures and Tables

**Table 2:** Summary of key *Candidozyma auris* antifungal resistance mechanisms.

Resistance Mechanism	Drug Class Affected	Key Genes/ Pathways	Mechanistic Basis	References
Target alteration	Azoles	*ERG11*	Mutations alter lanosterol 14-α-demethylase, reducing azole binding and sterol pathway inhibition	[106,107]
Efflux pump overexpression	Azoles (primarily)	*CDR1*, *MDR1*, *TAC1B*	Increased drug export from fungal cells, leading to reduced intracellular drug concentration	[108,109]
Echinocandin target mutations	Echinocandins	*FKS1* hotspot mutations (S639, etc.)	Reduced affinity of β-1,3-glucan synthase for echinocandins	[7,36]
Biofilm-mediated resistance	Multiple classes	Biofilm matrix genes	Extracellular matrix and altered physiology reduce drug penetration and increase tolerance	[31,101]
Stress response pathways	Broad tolerance	Hsp90, Hog1 MAPK	Cellular stress signaling enhances survival under antifungal pressure	[18,24,89]
Chromosomal duplication/genomic plasticity	Azoles/multidrug	*ERG11* amplification, aneuploidy	Gene dosage effects increase resistance-associated protein expression	[110]
Phenotypic heterogeneity/heteroresistance	Multiple classes	population-level variability	Subpopulations transiently tolerate antifungals without stable genetic resistance	[111]

**Table 3:** *Candidozyma auris* tentative breakpoints by CLSI and EUCAST *. Values are in mg/L.

	CLSI	EUCAST
Fluconazole	R ≥ 32	Not reported
Amphotericin B	R ≥ 2	ECOFF; S ≤ 0.001; R > 2
Caspofungin	R ≥ 2	Not reported
Micafungin	R ≥ 4	ECOFF: 2; S ≤ 0.25; R > 0.25
Anidulafungin	R ≥ 4	ECOFF: 2; S ≤ 0.25; R > 0.25
Rezafungin	Not reported	ECOFF: 0.125
5-Flucytosine	Not reported	ECOFF: 0.5

ECOFF, epidemiological cut-off values; S, susceptible; R, resistant. * Source: Centers for Disease Control and Prevention, National Center for Emerging and Zoonotic Infectious Diseases (NCEZID), Division of Foodborne, Waterborne, and Environmental Diseases (DFWED), and European Committee for Antimicrobial Testing [123,128,129].
